# Exposure to a Mixture of Endocrine-Disrupting Chemicals and Metabolic Outcomes in Belgian Adolescents

**DOI:** 10.1021/acs.est.3c07607

**Published:** 2023-11-09

**Authors:** Anran Cai, Sylvie Remy, Virissa Lenters, Bianca Cox, Greet Schoeters, Adrian Covaci, Roel Vermeulen, Lützen Portengen

**Affiliations:** † Institute for Risk Assessment Sciences, Department of Population Health Sciences, Utrecht University, Utrecht 3584 CM, The Netherlands; ‡ VITO Health, Flemish Institute for Technological Research (VITO), Mol 2400, Belgium; § Amsterdam Institute for Life and Environment, Department of Environment and Health, Vrije Universiteit Amsterdam, Amsterdam 1081 HV, The Netherlands; ∥ Department of Biomedical Sciences, University of Antwerp, Antwerp 2000, Belgium; ⊥ Toxicological Centre, University of Antwerp, Wilrijk 2610, Belgium; # Julius Center for Health Sciences and Primary Care, University Medical Center Utrecht, Utrecht 3584 CG, The Netherlands

**Keywords:** endocrine-disrupting chemicals, body mass index, abdominal obesity, cholesterol, triglycerides, adolescence

## Abstract

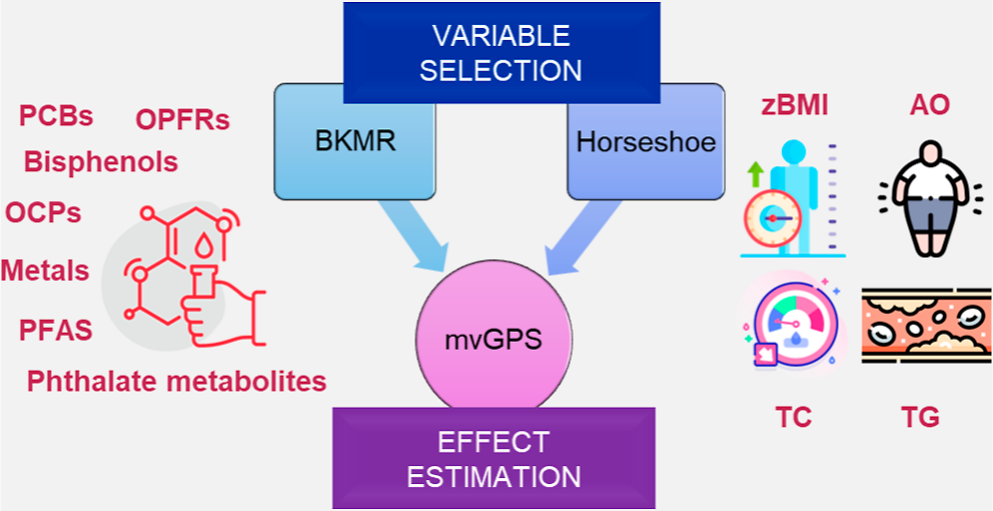

Childhood exposure to endocrine-disrupting chemicals (EDCs), either alone or in mixtures, may affect metabolic outcomes, yet existing evidence remains inconclusive. In our study of 372 adolescents from the Flemish Environment and Health Study (FLEHS IV, 2017–2018), we measured 40 known and suspected EDCs and assessed metabolic outcomes, including body mass index *z*-score (zBMI), abdominal obesity (AO), total cholesterol (TC), and triglycerides (TG). We applied Bayesian kernel machine regression (BKMR) and Bayesian penalized horseshoe regression for variable selection and then built multivariate generalized propensity score (mvGPS) models to provide an overview of the effects of selected EDCs on metabolic outcomes. As a result, BKMR and horseshoe together identified five EDCs associated with zBMI, three with AO, three with TC, and five with TG. Through mvGPS analysis, monoiso-butyl phthalate (MIBP), polychlorinated biphenyl (PCB-170), and hexachlorobenzene (HCB) each showed an inverse association with zBMI, as did PCB-170 with AO. Copper (Cu) was associated with higher TC and TG, except in boys where it was linked to lower TG. Additionally, monoethyl phthalate (MEP) and monobenzyl phthalate (MBzP) were associated with higher TG. To conclude, our findings support the association between certain chemicals (Cu, MEP, and MBzP) and elevated lipid levels, aligning with prior studies. Further investigation is needed for sex-specific effects.

## Abstract 2

Humans are exposed to numerous environmental chemicals and mixture assessment can be challenging. This study applied a novel statistical framework to comprehensively examine the potential metabolic health risks associated with chemical mixtures in early life.

## 1 Introduction

Childhood, including adolescence, is a vulnerable period when it comes to exposure to environmental chemicals and their mixtures.^1^ Many of these chemicals are endocrine-disrupting chemicals (EDCs) since they can interfere with the hormonal system in the body, and exposure to these chemicals during critical developmental stages may lead to adverse health effects later in life.^2^ EDCs are widely present in the environment, including both persistent chemicals such as organochlorinated pesticides (OCPs), polychlorinated biphenyls (PCBs), per- and polyfluoroalkyl substances (PFAS), some metals, and nonpersistent chemicals such as organophosphate flame retardants (OPFRs), bisphenols, and phthalates. Their exposures can occur through ingestion, inhalation, or absorption through the skin.^3^

Metabolic outcomes, such as sex- and age-specific body mass index *z*-score (zBMI), abdominal obesity (AO), total cholesterol (TC), and triglyceride (TG) levels, are commonly used as important indicators to assess the risk of developing chronic diseases, including cardiovascular disease, type 2 diabetes, and metabolic syndrome. zBMI is a body measure relative to an individual’s weight and height, with values higher than 1 standard deviation (SD) above the WHO Growth Reference median being considered as overweight or obese for children aged between 5 and 19 years.^4^ AO is particularly hazardous because the fat accumulated in the abdomen is metabolically active, and can release substances that contribute to inflammation and insulin resistance.^5,6^ TC level is a measure of the amount of cholesterol in the blood, including both high-density lipoprotein (HDL) cholesterol and low-density lipoprotein (LDL) cholesterol. Higher levels of TC and LDL cholesterol are associated with an increased risk of heart disease and stroke.^7^ TG are lipids found in the blood that can increase the risk of hypothyroidism and heart disease when levels are too high.^8^

Exposure to EDCs can lead to changes in metabolic outcomes by disrupting hormone signaling, promoting inflammation, increasing oxidative stress, and altering the composition of the gut microbiota.^9^ Epidemiological studies have been instrumental in determining the potential health effects of exposure to EDCs on metabolic outcomes in adolescents.^10−18^ However, the current evidence is inconclusive and the majority of these studies have focused on individual chemicals ignoring coexposures, despite the fact that humans are exposed to multiple chemicals simultaneously, and mixtures can have synergistic or antagonistic effects on human health that may be different from the effects of individual chemicals.^19^ The human body also metabolizes these chemicals in complex ways that can result in a variety of metabolites with different mechanisms of toxicity, leading to different health outcomes. Hence, a traditional single-exposure model that does not consider other exposures may not capture the complexity of real-life risks, and there is growing interest in using advanced statistical methods to investigate the effects of EDC mixtures on metabolic outcomes.

Understanding the relationship between EDC mixtures and metabolic outcomes is crucial in policy-making to develop effective prevention of chronic diseases. Using a cross-sectional design, we studied the real–world associations between a large mixture of known and suspected EDCs (OCPs, PCBs, PFAS, metals, OPFRs, bisphenols, and phthalate metabolites),^20−22^ and several metabolic outcomes (zBMI, AO, TC, and TG levels) in Belgian adolescents.

## 2 Methods

### 2.1 Study Design and Population

We used data from the fourth campaign of the Flemish Environment and Health Study (FLEHS IV, 2017–2018), a sample of 428 adolescents aged 14 to 15 years living in the Flemish region of Belgium. The details of the sampling strategy have been described previously.^23^ Briefly, adolescents who had lived in Flanders for at least five years and were able to fill out a questionnaire in Dutch were eligible to participate. The selection process employed a stratified clustered two-stage sampling design, where the first stratification was with Flemish provinces followed by a random selection of schools from each province. As a result, the participation rate was proportional to the population size of each province. The schools chosen were at least 20 km apart, and within each province, one school from the highest quartile of socially deprived attendees was included to ensure representation from all socio-economic categories. Exclusions from participation encompassed individuals with more than one unanswered questionnaire, missing blood or urine samples, experiencing retention of one or more years in their school grade, attending a boarding school, or being pregnant. Approval for the FLEHS IV study protocol was granted in June 2017 by the Ethics Committee of Antwerp University Hospital (Belgian registration number B300201732753). The current study was restricted to adolescents who had all measurements available for the relevant exposures, outcomes, and covariates, resulting in a total of 372 adolescents.

### 2.2 Exposure Assessment

To account for the complex and often unknown ways that multiple chemicals can interact to affect human metabolic health, we intended to explore a wide range of substances. An extensive set of chemicals have been measured in FLEHS IV. Of these chemicals, we included 40 suspected and established EDCs with a detection rate above 60% for this study.^24^ In detail, they were measured in blood (metals, OCPs, PCBs, PFAS) and urine samples (OPFRs, bisphenols, phthalate metabolites) (Table S1): two OCPs [dichloro-diphenyl-dichloroethylene (DDE), hexachlorobenzene (HCB)], six PCB congeners [PCB-118, PCB-138, PCB-153, PCB-170, PCB-180, PCB-187], four PFAS [perfluorooctanoic acid (PFOA), perfluorononan-1-oic acid (PFNA), perfluoro-1-hexanesulfonate (PFHxS), perfluorooctanesulfonate (PFOS)], six metals [cadmium (Cd), thallium (Tl), lead (Pb), manganese (Mn), copper (Cu), zinc (Zn)], five OPFR metabolites [bis(1,3-dichloro-2-propyl) phosphate (BDCIPP), diphenyl phosphate (DPHP), 1-hydroxy-2-propyl bis(1-chloro-2-propyl) phosphate (BCIPHIPP), 2-ethylhexyl phenyl phosphate (EHPHP), 2-hydroxyethyl bis(2-butoxyethyl) phosphate (BBOEHEP)], three bisphenols [bisphenol A (BPA), bisphenol F (BPA), bisphenol S (BPS)], and fourteen phthalate and other plasticizer metabolites [monoethyl phthalate (MEP), monoiso-butyl phthalate (MIBP), mononormal-butyl phthalate (MnBP), monobenzyl phthalate (MBzP), mono-(2-ethyl-5-carboxypentyl) phthalate (5-cx-MEPP), mono-(2-ethyl-5-hydroxyhexyl) phthalate (MEHHP), mono-(2-ethyl-5-oxohexyl) phthalate (5-oxo-MEHP), mono-(2-ethylhexyl) phthalate (MEHP), mono(2-ethyl-5-hydroxyhexyl) terephthalate (OH-MEHTP), OH-monoisononyl phthalate (OH-MINP), carboxy-mono octyl phthalate (cx-MINP), OH-monohydroxy-isodecyl phthalate (OH-MIDP), monocarboxy-isodecyl phthalate (cx-MIDP), mono-oxo-isodecyl phthalate (oxo-MIDP)]. A detailed description of exposure assessment was provided elsewhere.^23^ In short, spot urine and nonfasting blood samples were stored at −20 and −80 °C, respectively, until the applicable chemicals were measured. Metals were measured by high-resolution inductively coupled plasma mass spectrometry (HR-ICP-MS), OPFRs and phthalate metabolites by liquid chromatography with tandem mass spectrometry (LC–MS/MS), OCPs and PCBs by gas chromatograph with electron-capture negative ionization mass spectrometry (GC-ECNI/MS), bisphenols by gas chromatography with tandem mass spectrometry (GC–MS/MS), and PFAS by ultrahigh-performance liquid chromatography with tandem mass spectrometry (UPLC-MS/MS).^11,23,25−27^ Concentration values below the limits of detection (LODs) or limits of quantification (LOQs) were imputed using maximum likelihood estimation, assuming a censored log–normal distribution for values above the LODs or LOQs and conditional on the observed values for other biomarkers in the cohort.^24,28^ Lipid-soluble chemicals DDE, HCB, and PCBs were standardized by total blood lipid concentration [total lipids = 1.33 × TG + 1.12 × TC × 148 (g/L)] and their concentrations were therefore expressed in ng/g lipid. Urinary exposure concentrations were normalized for the specific gravity (SG) of the urine sample using the following formula: *C*_SG_ = *C*_exposure_ × (1.024 – 1)/(SG – 1).

### 2.3 Outcome Assessment

We assessed four metabolic outcomes of interest: zBMI, calculated based on an individual’s weight, height, and the World Health Organization (WHO) reference curves;^4^ AO, defined by waist-to-height ratio ≥ 0.5;^29^ as well as TC and TG levels measured from blood samples. Trained field staff conducted clinical measurements of height, weight, and waist circumference with participants fully clothed, excluding shoes.

### 2.4 Covariates

Using a directed acyclic graph (DAG; Figure S1), we identified a set of covariates to be adjusted for in the statistical analyses: sex (boy, girl), age (years), sampling season (spring, autumn, winter), ever breastfed (yes, no), highest education in the household [low (lower secondary school or less), medium (higher secondary school), high (higher education attainment)], and physical activity (never or rarely, 1–2 times a week, 3 or more times a week involved in sports). Information on all of those variables was obtained from the questionnaires filled out by participants and parents before the clinical measurements.

### 2.5 Statistical Analysis

We used the geometric mean, median, or frequency (%) to describe demographic characteristics as well as the distributions of exposures and outcomes. We calculated Pearson correlation coefficients between exposures and between outcomes. In order to improve the comparability and model fits, all exposures were centered and scaled to have a mean of 0 and SD of 1 for all of the analyses, and all the obtained effect estimates were expressed in β or odds ratio (OR) per SD of exposure.

We employed two Bayesian variable selection methods, namely, Bayesian kernel machine regression (BKMR) and Bayesian penalized (horseshoe) regression, to identify the main contributors among the 40 EDCs for the examined outcomes. These methods were chosen for their ability to handle high-dimensional and correlated data and effectively identify relevant variables while simultaneously controlling for overfitting. BKMR is a supervised nonparametric flexible method that allows for nonlinear exposure-outcome association and interaction between exposures.^30,31^ Its Bayesian framework enables estimation of the posterior distribution of regression coefficients for each exposure, providing a measure of their importance with respect to the outcome, as well as estimation of the overall mixture effect, i.e., the change in outcome if all exposures were fixed at incrementally higher quantiles. In this study, most exposures were only weakly correlated with other exposures, but some were correlated more strongly (Figure S2). Based on the correlation patterns and information on chemical properties, we grouped exposures within a same chemical class with a moderate-to-high correlation (*r* > 0.60) into a single group. This resulted in a total of 25 exposure groups for the hierarchical variable selection (Table S2). Then BKMR models were fitted using 4 chains and 50,000 iterations per chain and checked for convergence by visual inspection of trace plots. The embedded hierarchical variable selection provided the models with information about those 25 exposure groups to obtain an estimate of the relative importance of each group (group posterior inclusion probabilities, GroupPIP) and each exposure therein (conditional PIP, CondPIP). With regard to horseshoe regression, the “horseshoe” in the name refers to the shape of the shrinkage prior distribution used in this method. The horseshoe prior is designed to be particularly effective when dealing with situations where there might be a large number of irrelevant variables but a few truly significant ones. In this study, this method was employed to help identify important exposures by retaining their coefficients while shrinking irrelevant exposure coefficients toward zero. Following the recommendations for the horseshoe prior and hyperparameter settings from previous studies,^32,33^ we left the degrees of freedom (df) of the global and local shrinkage parameters at the default value of 1, and the df of the regularization parameter at the default value of 4. The scale prior to the regularization parameter was set to 2.5. We increased the parameter adapt_delta to 0.999 to avert divergent transitions. The combination of those two methods effectively identified sparse signals from a mixture and increased the confidence of the variable selection.

The selected exposures, determined by the aforementioned variable selection methods, were included as inputs in the multivariate generalized propensity score (mvGPS) models, which facilitated us to better estimate the effects of the selected exposures on the outcomes of interest and provided an overview. mvGPS is an extension of the generalized propensity score to estimate weights for multivariate continuous exposures simultaneously.^34^ It assumes a multivariate normal distribution for multiple exposures thereby generating stabilized inverse probability treatment weights (IPTWs) as a way to balance confounders and exposures and so provide unbiased causal effect estimates.^34,35^ During the propensity score weighting procedure, we used a default upper bound of 0.99, which was shown to be effective in trimming the weights.^36^ In addition, we conducted sex-stratified analyses on the selected exposures to evaluate the possible effect modification by sex.

As a sensitivity analysis, we refitted BKMR models without the hierarchy (i.e., component-wise variable selection) to explore different modeling assumptions, as component-wise variable selection assumes that each exposure has an independent effect, whereas hierarchical variable selection assumes that there may be shared effects among exposures within the same group. In another sensitivity analysis, given that current smoking is a potential confounder but the prevalence of smoking in our study population was too low (4.8%), we did not include smoking as a covariate in our main analyses and instead conducted a separate analysis that excluded adolescents who smoked.

All statistical analyses were performed in R 4.2.2.^37^ Horseshoe, BKMR and mvGPS were fitted using *brms*, *bkmrhat*, and *mvGPS* packages, respectively.^31,34,38^

## 3 Results

### 3.1 Descriptive Statistics

The sociodemographic and clinical characteristics of the adolescents included in this study are provided in Table 1. Of the 372 participants, 48% were boys and 52% were girls. Most participants had a member in their household with high education (60%), were ever breastfed (66%), and did not have AO (88%). Half of them performed physical activity 3 or more times a week. They were sampled in either spring (45%), autumn (22%), or winter (33%). On average, the participants were 14.8 years of age, had a zBMI of 0.2, and had TC and TG levels of 155.8 and 85.4 mg/dL, respectively. The three continuous metabolic outcomes were weakly correlated with each other (*r* = 0.04–0.17). The concentrations of the 40 EDCs spanned a wide range and are stated in Table S1. Some of them (e.g., PFAS, PCBs, and certain phthalate metabolites) were moderately to highly correlated (*r* > 0.60) while the rest had lower correlations with each other (Figure S2).

**Table 1:** Sociodemographic Characteristics and Metabolic Outcomes among FLEHS IV Adolescents (*n* = 372)^a^

Characteristic	Value
Age (years), mean	14.8
Sex, *n* (%)	
boy	177 (48)
girl	195 (52)
Highest Education in the Household, *n* (%)	
low	22 (6)
medium	125 (34)
high	225 (60)
Sampling Season, *n* (%)	
spring	168 (45)
autumn	83 (22)
winter	121 (33)
Physical Activity, *n* (%)	
never or rarely	52 (14)
1–2 times a week	134 (36)
3 or more times a week	186 (50)
Ever Breastfed, *n* (%)	
no	126 (34)
yes	246 (66)
Current Smoking, *n* (%)	
no	354 (95)
yes	18 (5)
zBMI, mean	0.2
TC (mg/dL), mean	155.8
TG (mg/dL), mean	85.4
AO, *n* (%)	
no	328 (88)
yes	44 (12)

a zBMI, body mass index *z*-score; AO, abdominal obesity; TC, total cholesterol; TG, triglycerides.

### 3.2 Overall Mixture Effects and Exposure Variable Selection

**Figure 1:**
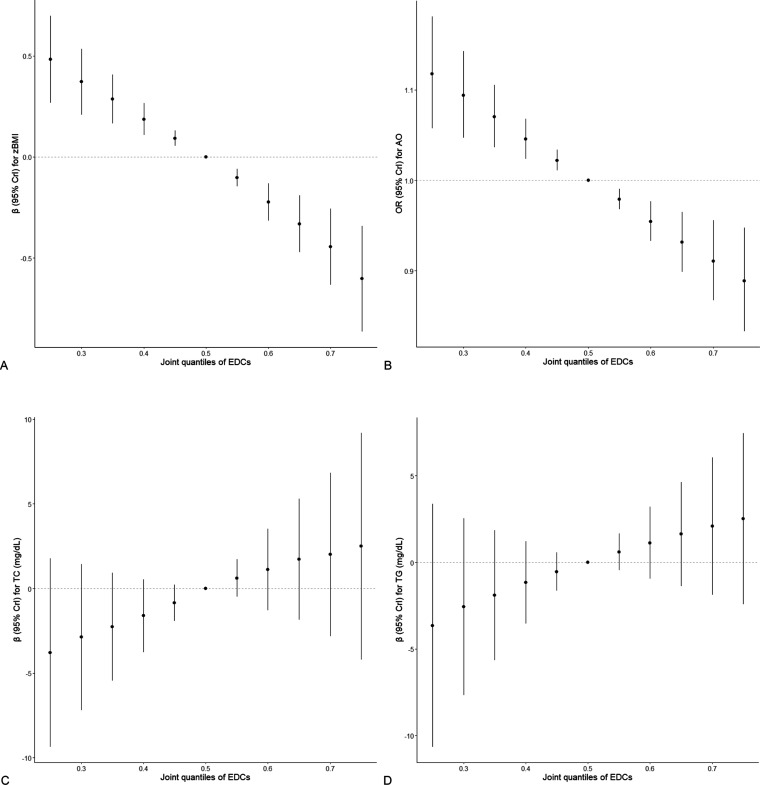
Overall mixture effects and 95% credible intervals (Crls) of endocrine-disrupting chemicals (EDCs) on metabolic outcomes [(A) body mass index *z*-score (zBMI), (B) abdominal obesity (AO), (C) total cholesterol levels (TC), (D) triglycerides (TG) levels], estimated using Bayesian kernel machine regression (BKMR). Note: models were adjusted for sex (except when the outcome was zBMI), age (except when the outcome was zBMI), sampling season, ever breastfed, highest education in the household, and physical activity.

Using BKMR, we observed an overall inverse trend of the EDC mixture with zBMI and AO, and an overall positive trend with TC and TG levels (Figure 1). The univariate exposure–response functions showed that most EDCs had a linear relationship with metabolic outcomes, but a few nonlinear relationships were also observed (Figure S3). The bivariate exposure–response functions displayed the effect on a metabolic outcome of exposure 1 when exposure 2 was fixed at its 10th, 50th, or 90th percentiles and all remaining exposures were fixed at their median (Figure S4). However, interpretation of exposure interactions was limited due to inevitable sparsity issues that arose in pairwise interaction surface plots given the large number of exposures in the mixture. Finally, associations with metabolic outcomes were observed for certain EDCs identified as noteworthy contributors (Table 2): BDCIPP, HCB, PCB-170, and MIBP for zBMI; BPF, PCB-170, and MEHHP for AO; Cu and BBOEHEP for TC levels; Cu, HCB, MBzP, MEP, and MIBP for TG levels. This selection depended on the following criteria: when a chemical was the only exposure of a group, its group PIP exceeded 0.5; when a chemical was one of the multiple exposures of a group, its group PIP and CondPIP both exceeded 0.5.

**Table 2:** Posterior Inclusion Probabilities for Group and Conditional Inclusions into Metabolic Outcome Models, Using Bayesian Kernel Machine Regression (BKMR) Hierarchical Variable Selection^a^

Exposure (*n* = 40)	Exposure group used in analysis (*n* = 25)	zBMI		AO		TC		TG	
		GroupPIP^b^	CondPIP^c^	GroupPIP	CondPIP	GroupPIP	CondPIP	GroupPIP	CondPIP
Cd	1	0.04		0.08		0.05		0.29	
Tl	2	0.45		0.01		0.07		0.24	
Pb	3	0.08		0.01		0.01		0.16	
Mn	4	0.15		0.23		0.03		0.35	
Cu	5	0.05		0.01		**0.58**		**0.68**	
Zn	6	0.02		0.02		0.23		0.27	
BDCIPP	7	**0.79**		0.01		0.08		0.15	
DPHP	8	0.09		0.01		0.06		0.03	
BCIPHIPP	9	0.12		0.04		0.07		0.32	
EHPHP	10	0.13		0.25		0.04		0.14	
BBOEHEP	11	0.01		0.02		**0.83**		0.11	
DDE	12	0.12		0.03		0.1		0.14	
HCB	13	**0.99**		0.12		0.04		**0.79**	
PCB-118	14	1	0	0.99	0	0.07	0.21	0.29	0.15
PCB-138	14	1	0	0.99	0.17	0.07	0.17	0.29	0.15
PCB-153	14	1	0.25	0.99	0.07	0.07	0.15	0.29	0.15
PCB-170	14	**1**	**0.75**	**0.99**	**0.72**	0.07	0.16	0.29	0.17
PCB-180	14	1	0	0.99	0.03	0.07	0.09	0.29	0.22
PCB-187	14	1	0	0.99	0.01	0.07	0.22	0.29	0.16
BPA	15	0.34		0.05		0.04		0.06	
BPF	16	0.11		**0.99**		0.08		0.11	
BPS	17	0.32		0.01		0.05		0.14	
PFOA	18	0.18	0.05	0.04	0.14	0.14	0.72	0.03	0.26
PFNA	18	0.18	0.09	0.04	0.79	0.14	0.13	0.03	0.21
PFHSX	18	0.18	0.83	0.04	0	0.14	0.11	0.03	0.30
PFOS	18	0.18	0.03	0.04	0.07	0.14	0.05	0.03	0.22
MEP	19	0.01		0.53		0.05		**0.62**	
MIBP	20	**0.64**		0.01		0.05		**0.58**	
MnBP	21	0.03		0.02		0.01		0.17	
MBzP	22	0.15		0.41		0.04		**0.64**	
5cx-MEPP	23	0.05	0.23	0.98	0.09	0.05	0.28	0.05	0.25
MEHHP	23	0.05	0.27	**0.98**	**0.67**	0.05	0.19	0.05	0.25
5oxo-MEHP	23	0.05	0.34	0.98	0.24	0.05	0.2	0.05	0.25
MEHP	23	0.05	0.16	0.98	0.01	0.05	0.33	0.05	0.25
OH-MEHTP	24	0.21	0.44	0.01	0.52	0.23	0.74	0.46	0.52
OH-MINP	24	0.21	0.56	0.01	0.48	0.23	0.26	0.46	0.48
cx-MINP	25	0.09	0.14	0.03	0.2	0.05	0.1	0.13	0.23
OH-MIDP	25	0.09	0.52	0.03	0.26	0.05	0.2	0.13	0.29
cx-MIDP	25	0.09	0.15	0.03	0.36	0.05	0.38	0.13	0.22
oxo-MIDP	25	0.09	0.19	0.03	0.18	0.05	0.32	0.13	0.26

a zBMI, body mass index *z*-score; AO, abdominal obesity; TC, total cholesterol; TG, triglycerides. Note: numbers in bold refer to ones with GroupPIP > 0.5 (when there is only one chemical in a group), or with both GroupPIP and CondPIP > 0.5 (when there is more than one chemical in a group).

b GroupPIP indicates the posterior probability that an exposure group was included in the BKMR model from the Markov chain Monte Carlo (MCMC) sampler.

c CondPIP indicates the posterior probability that a particular exposure within an exposure group was included in the BKMR model from the MCMC sampler.

**Figure 2:**
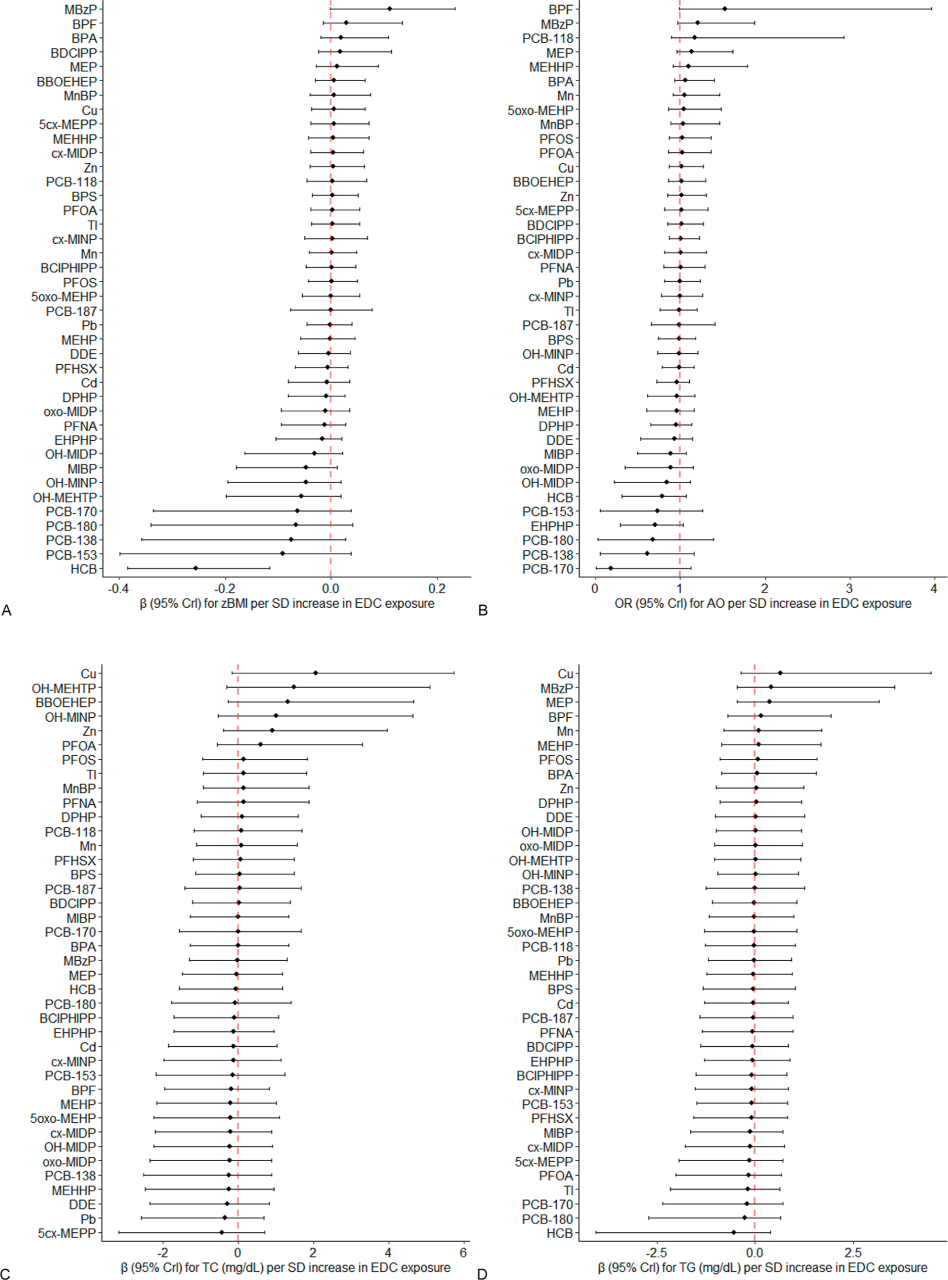
Point estimates and 95% credible intervals (Crls) of metabolic outcomes [(A) body mass index *z*-score (zBMI), (B) abdominal obesity, (C) total cholesterol levels, (D) triglycerides levels] per standard deviation increase in EDC exposures, evaluated with penalized horseshoe regression. Note: models were adjusted for sex (except when the outcome was zBMI), age (except when the outcome was zBMI), sampling season, ever breastfed, highest education in the household, and physical activity.

Through the application of horseshoe regression, we identified several exposures that exhibited the strongest associations with specific metabolic outcomes. MBzP demonstrated a notable positive association, whereas HCB showed an inverse association with zBMI (Figure 2A); BPF was positively associated with AO, and PCB-170 showed a protective effect against it (Figure 2B). Additionally, per SD increase in Cu, OH-MEHTP, and BBOEHEP demonstrated the positive associations with TC levels, with estimated βs [mg/dL, 95% credible intervals (Crls)] of 2.00 (−0.14, 5.58), 1.48 (−0.29, 5.16), 1.32 (−0.24, 4.57), respectively (Figure 2C); Cu was suggested as the main EDCs contributing to higher TG levels with an estimated β (95% CrI, mg/dL) of 0.65 (−0.38, 4.41) (Figure 2D).

### 3.3 Effects of Selected Exposures on Metabolic Outcomes

Table 3 presents results from the mvGPS analysis. All chemical exposures selected from BKMR and horseshoe regression were included in the mvGPS models. Per SD increase in MIBP [β = −0.18, 95% confidence interval (CI): −0.31, −0.06], PCB-170 (β = −0.13, 95% CI: −0.22, −0.04), and HCB (β = −0.45, 95% CI: −0.58, −0.04) were linked to lower zBMI; PCB-170 (OR = 0.02, 95% CI: 0.004, 0.09) was related to a lower risk of AO. Cu (β = 5.02 mg/dL, 95% CI: 1.99, 8.05) showed an association with increased TC level, while MEP (β = 7.26 mg/dL, 95% CI: 3.85, 10.67), MBzP (β = 7.00 mg/dL, 95% CI: 2.19, 11.81) and Cu (β = 5.10 mg/dL, 95% CI: 0.94, 9.27) showed associations with increased TG levels. After stratification by child’s sex, most of the observed associations remained. Nevertheless, TG levels tended to increase only in girls but decreased in boys with Cu exposure.

**Table 3:** Estimated β or Odds Ratio (OR) of Metabolic Outcomes per Standard Deviation Increase in Selected EDC Exposures, Evaluated with Multivariate Generalized Propensity Score (mvGPS)^a^

	Exposure	Overall, *n* = 372	Boys, *n* = 177	Girls, *n* = 195
zBMI, β (95% CI)	BDCIPP	0.17 (−0.04, 0.37)	0.29 (−0.12, 0.71)	0.42 (0.03, 0.82)
	MBzP	0.09 (−0.04, 0.22)	0.17 (−0.07, 0.41)	0.01 (−0.19, 0.20)
	MIBP	–0.18 (−0.31, −0.06)	–0.38 (−0.65, −0.11)	–0.09 (−0.31, 0.13)
	PCB-170	–0.13 (−0.22, −0.04)	–0.33 (−0.51, −0.15)	–0.20 (−0.37, −0.03)
	HCB	–0.45 (−0.58, −0.04)	–0.38 (−0.55, −0.21)	–0.61 (−0.83, −0.39)
AO, OR (95% CI)	BPF	1.71 (0.71, 4.69)	2.17 (0.50, 11.88)	1.56 (0.16, 10.15)
	MEHHP	1.27 (0.89, 1.81)	1.06 (0.56, 2.00)	1.76 (0.91, 3.40)
	PCB-170	0.02 (0.004, 0.09)	0.01 (0.0004, 0.08)	0.04 (0.004, 0.23)
TC (mg/dL), β (95% CI)	Cu	5.02 (1.99, 8.05)	3.32 (−3.63, 10.26)	4.84 (1.76, 7.91)
	BBOEHEP	4.10 (−0.14, 8.35)	5.90 (−1.41, 13.21)	–2.47 (−9.69, 4.75)
	OH-MEHTP	–4.64 (−15.33, 6.05)	–13.46 (−30.8, 3.88)	–3.42 (−15.24, 8.41)
TG (mg/dL), β (95% CI)	MEP	7.26 (3.85, 10.67)	1.27 (−21.28, 23.82)	3.02 (−2.83, 8.87)
	MBzP	7.00 (2.19, 11.81)	3.53 (−5.88, 12.93)	5.87 (−0.81, 12.55)
	Cu	5.10 (0.94, 9.27)	–8.26 (−19.15, 2.62)	10.6 (7.38, 13.83)
	HCB	–5.35 (−11.98, 1.09)	–8.48 (−14.76, −2.21)	–5.3 (−12.48, 1.89)
	MIBP	–7.17 (−16.14, 1.80)	–8.78 (−20.99, 3.42)	–7.68 (−15.5, 0.13)

a zBMI, body mass index *z*-score; AO, abdominal obesity; TC, total cholesterol; TG, triglycerides; CI, confidence interval; OR, odds ratio. Note: models were adjusted for sex (except when the outcome was zBMI), age (except when the outcome was zBMI), sampling season, ever breastfed, highest education in the household, and physical activity.

### 3.4 Sensitivity Analysis

Minor variations were observed between the results of hierarchical variable selection and component-wise variable selection for BKMR model fits (Tables 2 and S3). Specifically, the latter approach selected two PCBs (PCB-153 and −170) for zBMI instead of just PCB-170 as in the former approach. Furthermore, when current smokers were excluded, negligible differences were observed (data not shown).

## 4 Discussion

The aim of this study was to determine whether exposure to EDCs was associated with metabolic health risks among adolescents enrolled in FLEHS IV. Through the investigation of anthropometric measures and lipid profiles, we found a decreasing trend of zBMI and AO with an overall mixture of 40 EDCs, whereas an increasing trend of TC and TG. More importantly, several specific EDCs were identified to have detrimental effects that warrant attention and require further research to validate and understand the underlying mechanisms.

Consistent with our findings, a study of Iranian children and adolescents revealed that MBzP was associated with elevated TG levels,^39^ and a positive correlation between MEP and TG has been observed in Chinese adults.^40^ We found associations of Cu with increased TG and TC levels, which were in line with the results from a large population-based study of 27,576 participants in Canada.^41^ We also noticed possible sex difference, with an inverse relationship between Cu and TG levels in boys. Although reduced TG levels after dietary copper supplementation were found in male rats,^42^ no sex difference has been reported in previous epidemiological studies. We hypothesize that the reason for the sex difference we observed may be the different dietary habits among boys and girls and possibly the effects of estrogen on Cu metabolism, but it may also be due to insufficient statistical power due to the reduced sample size for sex-stratified analysis. To the best of our knowledge, this is the first time that the potential association of BBOEHEP and OH-MEHTP with TC has been explored in humans, which requires further validation. Nevertheless, rodent studies have shown a similar relationship between the parent compound of OH-MEHTP, di(2-ethylhexyl) terephthalate (DEHT), and reduced TC, aligning with our observations.^43,44^ Also, the parent compound of BBOEHEP, tris(2-butoxyethyl) phosphate (TBOEP), has previously been linked to altered lipid metabolism.^45^ Overall, it is important to highlight that several longitudinal studies have shown that high TC and TG levels during adolescence can track into adulthood, leading to an increased risk of developing cardiovascular disease later in life.^46,47^ Therefore, particular attention should be given to the screening and prevention of disrupted lipid profiles in adolescents, along with the biomonitoring of chemicals that could potentially be associated with these lipid profiles.

In two recent meta-analysis, associations between PCBs and BMI were inconclusive but HCB and MIBP were associated with higher BMI in childhood, which is contradictory to our findings.^13,48^However, given the cross-sectional design of our study, reverse causality may exist. As shown in a previous study of Belgian adolescents, there was an increase of 1.6–2.3% in PCBs and HCB levels per unit decrease in zBMI, which may be attributed to the fact that some chemicals are released from adipose tissues during periods of weight lose through lipid metabolism leading to higher circulating blood concentrations.^49^ Likewise, in line with our finding on higher PCB-170 levels with lower AO risk, another dietary intervention study in obese adults reported an inverse correlation between total PCBs and abdominal adiposity.^50^ Given that MIBP has a short half-life of approximately four hours, a single measurement of MIBP may primarily reflect recent or short-term exposure rather than long-term exposure patterns, and thus this imprecise assessment of exposure may result in an inaccurate inverse relationship between MIBP and zBMI observed in this study. Altogether, the mixture of short- and long-lived chemicals and the yet unknown sensitive window of exposure may hamper further interpretation.

We presented here a metabolic health study with one of the largest number of chemicals evaluated either separately or as a mixture. With the combination of several state-of-the-art statistical methods, we addressed the top concerns surrounding mixture studies, including multicollinearity among individual EDCs, as well as their collective impact, thus providing more accurate variable selection and precise effect estimates. The statistical framework of variable selection plus propensity score used in this study could potentially be used in causal inference estimation with an appropriate study design.^36^ By assessing multiple outcomes, we gained a more comprehensive understanding of metabolic health in adolescents, as each indicator offers distinct insights into different aspects of metabolic disruption, including overall obesity, central obesity, lipid profile, etc. Furthermore, several sensitivity analyses helped assess the stability and robustness of the results.

This study has certain limitations, including the possible exposure misclassification due to relying on a single spot-blood/urine sample and challenges in establishing causality given the nature of the cross-sectional design. For a comprehensive evaluation of the lasting effects of EDC exposures on metabolic health, it would be beneficial to incorporate repeated or longitudinal measurements of both exposures (especially those with short half-lives) and outcomes to accurately account for fluctuations in exposure timing and potential changes in outcomes over time. In addition, there may be residual confounders, notably unmeasured variables, such as dietary factors, with which the lipid profiles may fluctuate.

This study provides valuable insights on how environmental chemical exposures may affect metabolic health, informing potential future public health policies aimed at reducing exposure to certain chemicals such as Cu, MEP, and MBzP, with the goal of improving lipid profiles and preventing chronic diseases in vulnerable populations.

## Supporting Information Available

Tables describing the exposure assessment in detail, the clustered exposure groups, and the Bayesian kernel machine regression component-wise variable selection and figures representing the directed acyclic graph, exposure correlations, nonlinear exposure-outcome associations, and exposure interactions (PDF)
