# Supplementary material for: Exposure to a Mixture of Endocrine-Disrupting Chemicals and Metabolic Outcomes in Belgian Adolescents
Source: Environ Sci Technol. 2023 Nov 9;57(48):19871–80. doi: 10.1021/acs.est.3c07607 (PMC10702523; doi:10.1021/acs.est.3c07607)
Supplement: Supplementary file 1 — es3c07607_si_001.pdf [file es3c07607_si_001.pdf]

Exposure to a Mixture of Endocrine-Disrupting Chemicals and Metabolic Outcomes in Belgian Adolescents

Anran Cai[1,2,*], Sylvie Remy[2], Virissa Lenters[1,3], Bianca Cox[2], Greet Schoeters[4], Adrian Covaci[5], Roel Vermeulen[1,6], Lützen Portengen[1]

[1] Institute for Risk Assessment Sciences, Department of Population Health Sciences, Utrecht University, Utrecht, the Netherlands
[2] VITO Health, Flemish Institute for Technological Research (VITO), Mol, Belgium
[3] Amsterdam Institute for Life and Environment, Department of Environment and Health, Vrije Universiteit Amsterdam, Amsterdam, the Netherlands
[4] Department of Biomedical Sciences, University of Antwerp, Antwerp, Belgium
[5] Toxicological Centre, University of Antwerp, Wilrijk, Belgium
[6] Julius Center for Health Sciences and Primary Care, University Medical Center Utrecht, Utrecht, the Netherlands
* Corresponding author. Email address: a.cai@uu.nl

Summary: 16 pages, 3 tables, 4 figures

Table of contents

Table S1. Exposure concentrations measured in FLEHS IV adolescents (n=372)……………..……………… 3

Table S2. Clustered exposure groups used in the Bayesian kernel machine regression (BKMR) based on the chemical properties and the correlation between exposures (r>0.6) ……………………………..…………. 5

Table S3. Posterior inclusion probabilities (PIPs) into metabolic outcome models, using Bayesian kernel machine regression (BKMR) component-wise variable selection ………………………..……………………6

Figure S1. Directed acyclic graph of authors' conception of the associations between endocrine disrupting chemicals, covariates and metabolic outcomes. ……………………………………………………….….…..7

Figure S2. Pearson correlation coefficient matrix for forty endocrine disrupting chemicals …………………....8

Figure S3. Univariate exposure-response functions and 95% credible intervals for the change in metabolic outcomes ……………………….………………...9

Figure S4. Qualitative interaction assessment between exposure 1 and exposure 2 in response to metabolic outcomes …………...………………………………………………………………..………………………13

Table S1. Exposure concentrations measured in FLEHS IV adolescents (n=372).

Chemical class	Exposure	Method	LOD or LOQ (μg/L)	≥ LOD or LOQ, n(%)	GM	SD	P25, P75
Metals in blood, μg/L	Cd	HR-ICP-MS	LOD=0.007	372 (100)	0.2	0.2	0.1, 0.2
	Tl		LOD=0.001	372 (100)	0.03	0.02	0.02, 0.03
	Pb		LOD=0.048	372 (100)	7.7	3.9	6.0, 9.7
	Mn		LOD=0.12	372 (100)	9.4	2.8	7.8, 11.4
	Cu		LOD=0.551	372 (100)	814	186	719, 871
	Zn		LOD=8	372 (100)	5302	861	4775, 5933
OPFRs in urine (SG-normalized), μg/L	BDCIPP	LC-MS/MS	LOQ=0.05	294 (79)	0.3	4.0	0.1, 0.7
	DPHP		LOQ=0.1	369 (99)	1.4	1.9	0.9, 2.0
	BCIPHIPP		LOQ=0.04	354 (95)	0.7	9.7	0.3, 1.5
	EHPHP		LOQ=0.05	369 (99)	4.1	3.7	2.8, 6.5
	BBOEHEP		LOQ=0.005	356 (96)	0.04	0.1	0.02, 0.1
OCPs in serum (lipid-standardized), ng/g lipid	DDE	GC-ECNI/MS	LOQ=0.004	372 (100)	41.5	87.2	22.9, 63.4
	HCB		LOQ=0.002	372 (100)	7.6	3.7	5.8, 9.9
PCBs in serum (lipid-standardized), ng/g lipid	PCB-118	GC-ECNI/MS	LOQ=0.002	371 (99)	2.2	1.4	1.6, 2.9
	PCB-138		LOQ=0.002	372 (100)	7.0	4.9	4.7, 10.1
	PCB-153		LOQ=0.002	372 (100)	10.0	8.7	6.3, 15.5
	PCB-170		LOQ=0.002	371 (99)	2.1	2.4	1.3, 3.3
	PCB-180		LOQ=0.004	372 (100)	4.6	5.5	2.7, 7.2
	PCB-187		LOQ=0.002	330 (89)	1.0	1.3	0.7, 1.9
Bisphenols in urine (SG-normalized), μg/L	BPA	GC-MS/MS	LOQ=0.3	319 (86)	1.0	2.0	0.7, 1.9
	BPF		LOQ=0.02	360 (97)	0.2	1.9	0.1, 0.3
	BPS		LOQ=0.04	307 (83)	0.1	1.4	0.1, 0.2
PFAS in serum, μg/L	PFOA	UPLC-MS/MS	LOQ=0.2	372 (100)	1.0	0.4	0,8, 1.3
	PFNA		LOQ=0.2	308 (83)	0.3	0.2	0.2, 0.4
	PFHSX		LOQ=0.2	360 (97)	0.5	0.6	0.3, 0.7
	PFOS		LOQ=0.2	372 (100)	2.2	2.3	1.4, 3.2
Phthalates in urine (SG-normalized), μg/L	MEP	LC-MS/MS	LOQ=0.5	372 (100)	36.6	475	15.0, 66.2
	MIBP		LOQ=0.5	372 (100)	25.6	59.7	14.6, 41.7
	MnBP		LOQ=0.5	372 (100)	19.6	18.2	12.7, 31.2
	MBzP		LOQ=0.2	366 (98)	3.0	14.3	1.5, 5.7
	5cx-MEPP		LOQ=0.5	372 (100)	16.4	9.3	12.1, 21.9
	MEHHP		LOQ=0.2	371 (99)	6.7	10.4	4.1, 10.1
	5oxo-MEHP		LOQ=0.2	370 (99)	4.2	6.6	2.7, 6.6
	MEHP		LOQ=0.5	311 (84)	1.2	2.3	0.8, 2.1
	OH-MEHTP		LOQ=0.2	329 (88)	0.6	7.1	0.3, 1.0
	OH-MINP		LOQ=0.2	372 (100)	4.6	21.7	2.8, 6.4

	cx-MINP		LOQ=0.2	370 (99)	2.1	12.7	1.3, 2.9
	OH-MIDP		LOQ=0.2	343 (92)	0.8	3.3	0.4, 1.2
	cx-MIDP		LOQ=0.2	372 (100)	1.4	2.3	1.1, 1.6
	oxo-MIDP		LOQ=0.2	293 (79)	0.4	1.2	0.3, 0.7

LOD: limit of detection, LOQ: limit of quantification, GM: geometric mean, SD: standard deviation, OPFRs: organophosphate flame retardants, OCPs: organochlorinated chlorinated pesticides, PCBs: polychlorinated biphenyls, PFAS: per- and polyfluoroalkyl substances, HR-ICP-MS: high resolution Inductively coupled plasma mass spectrometry, LC-MS/MS: liquid chromatography with tandem mass spectrometry, GC-ECNI/MS: gas chromatography with electron capture negative ion mass spectrometry, GC-MS/MS: gas chromatography with tandem mass spectrometry, UPLC-MS/MS: ultra-high-performance liquid chromatography with tandem mass spectrometry, SG, specific gravity.

Table S2. Clustered exposure groups used in the Bayesian kernel machine regression based on the chemical properties and the correlation between exposures (r>0.6).

Chemical class	Exposure (n=40)	Exposure group used in analysis (n=25)
Metals	Cd	1
	Tl	2
	Pb	3
	Mn	4
	Cu	5
	Zn	6
OPFRs	BDCIPP	7
	DPHP	8
	BCIPHIPP	9
	EHPHP	10
	BBOEHEP	11
OCPs	DDE	12
	HCB	13
PCBs	PCB-118	14
	PCB-138	14
	PCB-153	14
	PCB-170	14
	PCB-180	14
	PCB-187	14
Bisphenols	BPA	15
	BPF	16
	BPS	17
PFAS	PFOA	18
	PFNA	18
	PFHSX	18
	PFOS	18
Phthalates	MEP	19
	MIBP	20
	MnBP	21
	MBzP	22
	5cx-MEPP	23
	MEHHP	23
	5oxo-MEHP	23
	MEHP	23
	OH-MEHTP	24
	OH-MINP	24
	cx-MINP	25
	OH-MIDP	25
	cx-MIDP	25
	oxo-MIDP	25

OPFRs: organophosphate flame retardants, OCPs: organochlorinated chlorinated pesticides, PCBs: polychlorinated biphenyls, PFAS: per- and polyfluoroalkyl substances.

Table S3. Posterior inclusion probabilities (PIPs) for metabolic outcome models using Bayesian kernel machine regression component-wise variable selection.

Exposure	zBMI	AO	TC	TG
Cd	0.01	0.06	0.02	0.21
Tl	0.35	0.01	0.02	0.29
Pb	0.06	0.002	0.02	0.17
Mn	0.14	0.19	0.02	0.15
Cu	0.04	0.01	**0.88**	**0.55**
Zn	0.01	0.08	0.17	0.17
BDCIPP	**0.65**	0.01	0.01	0.18
DPHP	0.11	0.01	0.01	0.18
BCIPHIPP	0.09	0.02	0.01	0.21
EHPHP	0.13	0.18	0.01	0.15
BBOEHEP	0.01	0.01	**0.71**	0.21
DDE	0.07	0.01	0.03	0.2
HCB	**0.96**	0.07	0.01	**0.51**
PCB-118	0.05	0.02	0.01	0.15
PCB-138	0.07	0.39	0.03	0.25
PCB-153	**0.79**	0.04	0.01	0.27
PCB-170	**0.54**	**0.71**	0.01	0.18
PCB-180	0.46	0.02	0.01	0.42
PCB-187	0.1	0.04	0.01	0.23
BPA	0.16	0.02	0.01	0.17
BPF	0.06	**0.99**	0.03	0.23
BPS	0.31	0.002	0.03	0.19
PFOA	0.06	0.01	0.06	0.15
PFNA	0.04	0.07	0.004	0.16
PFHSX	0.28	0.003	0.01	0.15
PFOS	0.05	0.01	0.01	0.14
MEP	0.01	0.36	0.02	**0.59**
MIBP	**0.54**	0.002	0.02	**0.51**
MnBP	0.01	0.02	0.002	0.13
MBzP	0.07	0.28	0.002	**0.54**
5cx-MEPP	0.03	0.16	0.004	0.21
MEHHP	0.01	**0.72**	0.01	0.16
5oxo-MEHP	0.02	0.28	0.001	0.17
MEHP	0.02	0.02	0.01	0.17
OH-MEHTP	0.1	0.001	0.06	0.19
OH-MINP	0.13	0.01	0.06	0.18
cx-MINP	0.06	0.03	0.02	0.19
OH-MIDP	0.17	0.01	0.01	0.3
cx-MIDP	0.01	0.04	0.02	0.18
oxo-MIDP	0.05	0.008	0.004	0.15

zBMI, body mass index z-score; AO, abdominal obesity; TC, total cholesterol; TG, triglycerides.
Note: numbers in bold refer to ones with PIP>0.5.

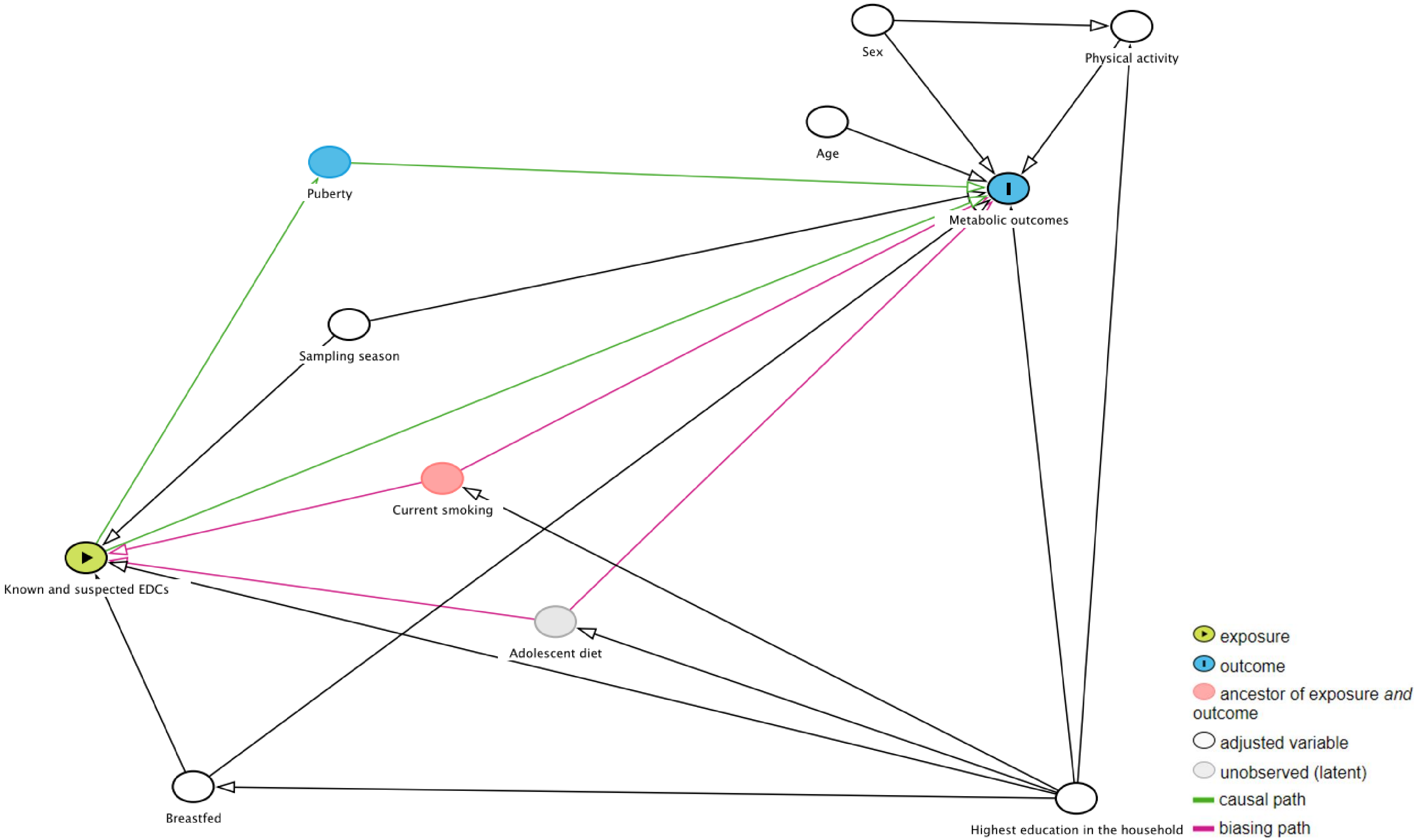

Figure S1. Directed acyclic graph of authors' conception of the associations between endocrine disrupting chemicals, covariates and metabolic outcomes. Note: sex and age were not adjusted when the outcome was body mass index z-score.

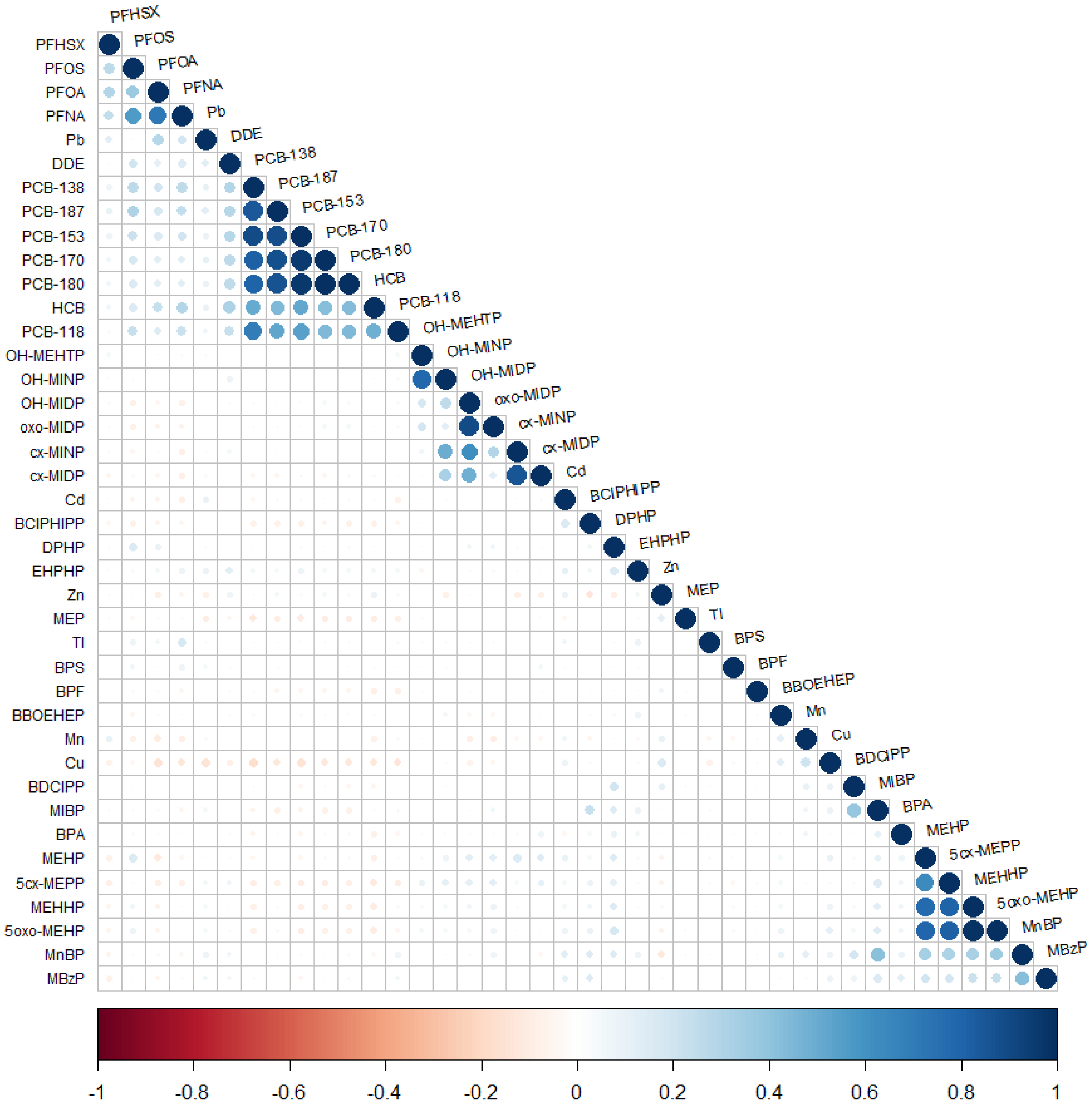

Figure S2. Pearson correlation coefficient matrix for forty endocrine disrupting chemicals. Note: the color intensity and the size of the circles indicate the strength of the pairwise correlation.

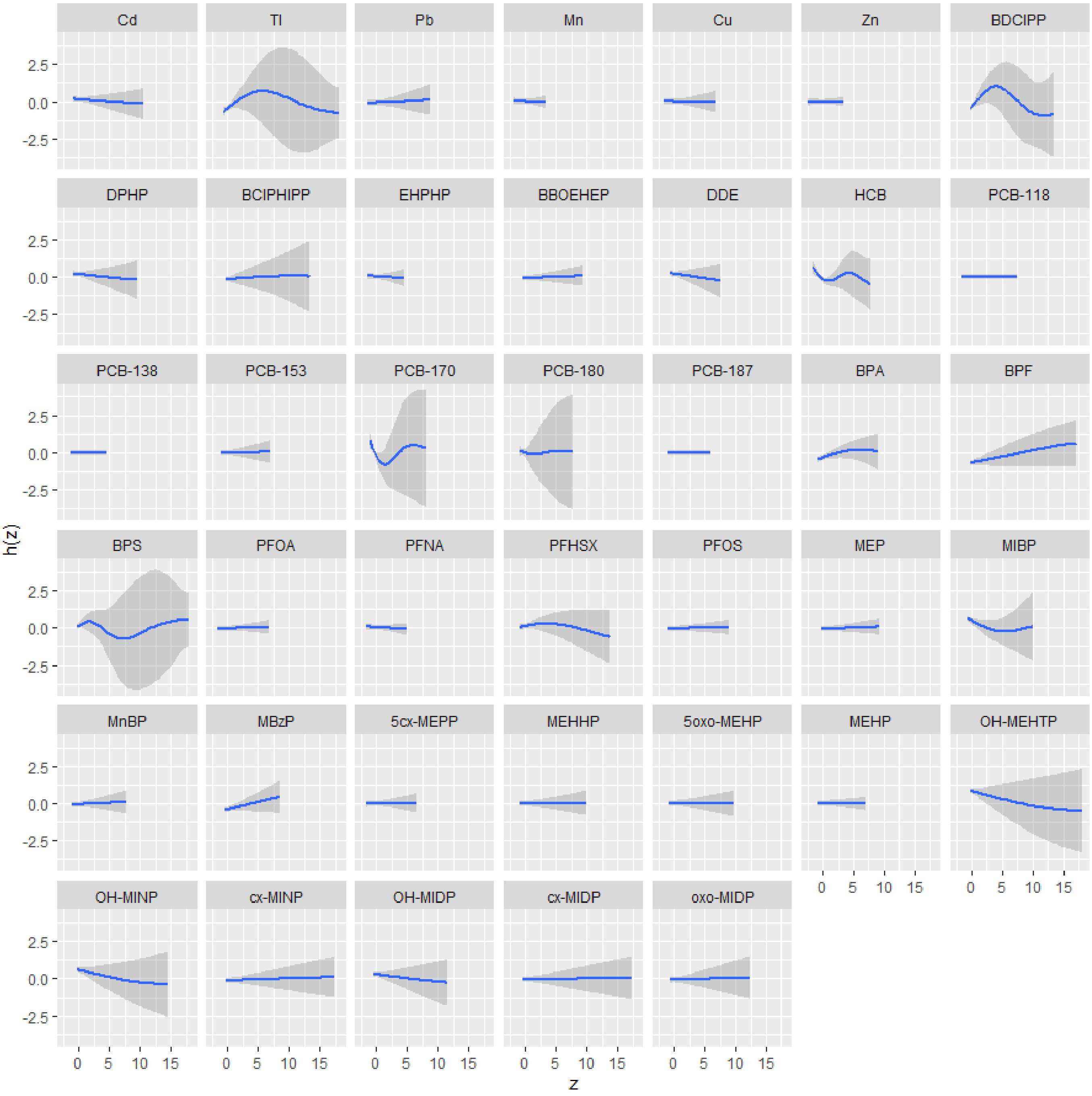

A

Figure S3. Univariate exposure-response functions and 95% credible intervals for the change in metabolic outcomes [A: body mass index z-score (zBMI), B: abdominal obesity (AO), C: total cholesterol levels (TC), D: triglycerides (TG) levels] from an endocrine disrupting chemical (EDC) while holding all the remaining EDCs in the mixture fixed at their median concentration, estimated using Bayesian kernel machine regression (BKMR).

Note: models were adjusted for sex (except when outcome was zBMI), age (except when outcome was zBMI), sampling season, ever breastfed, highest education in the household, and physical activity.

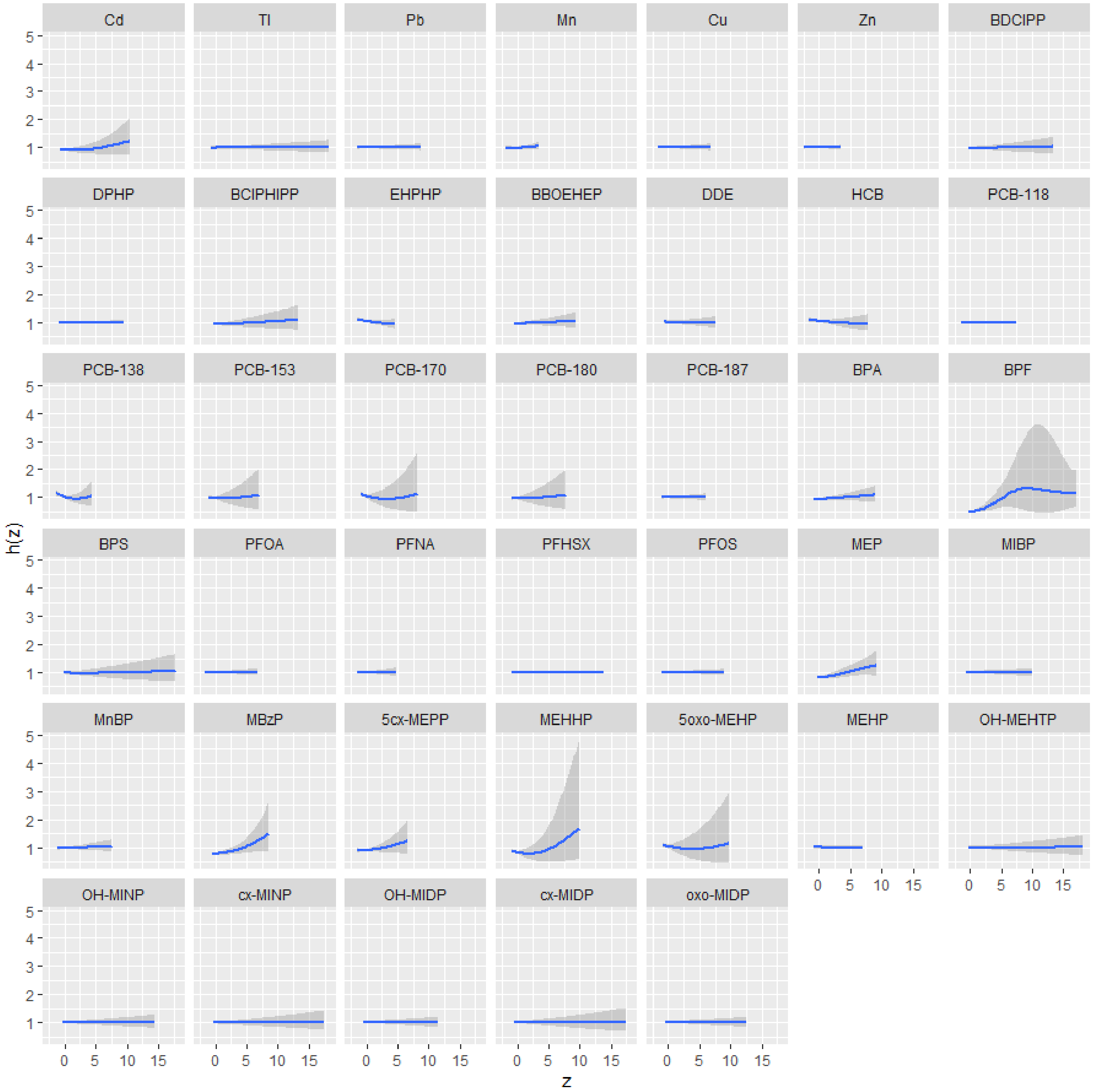

B

Figure S3 continued.

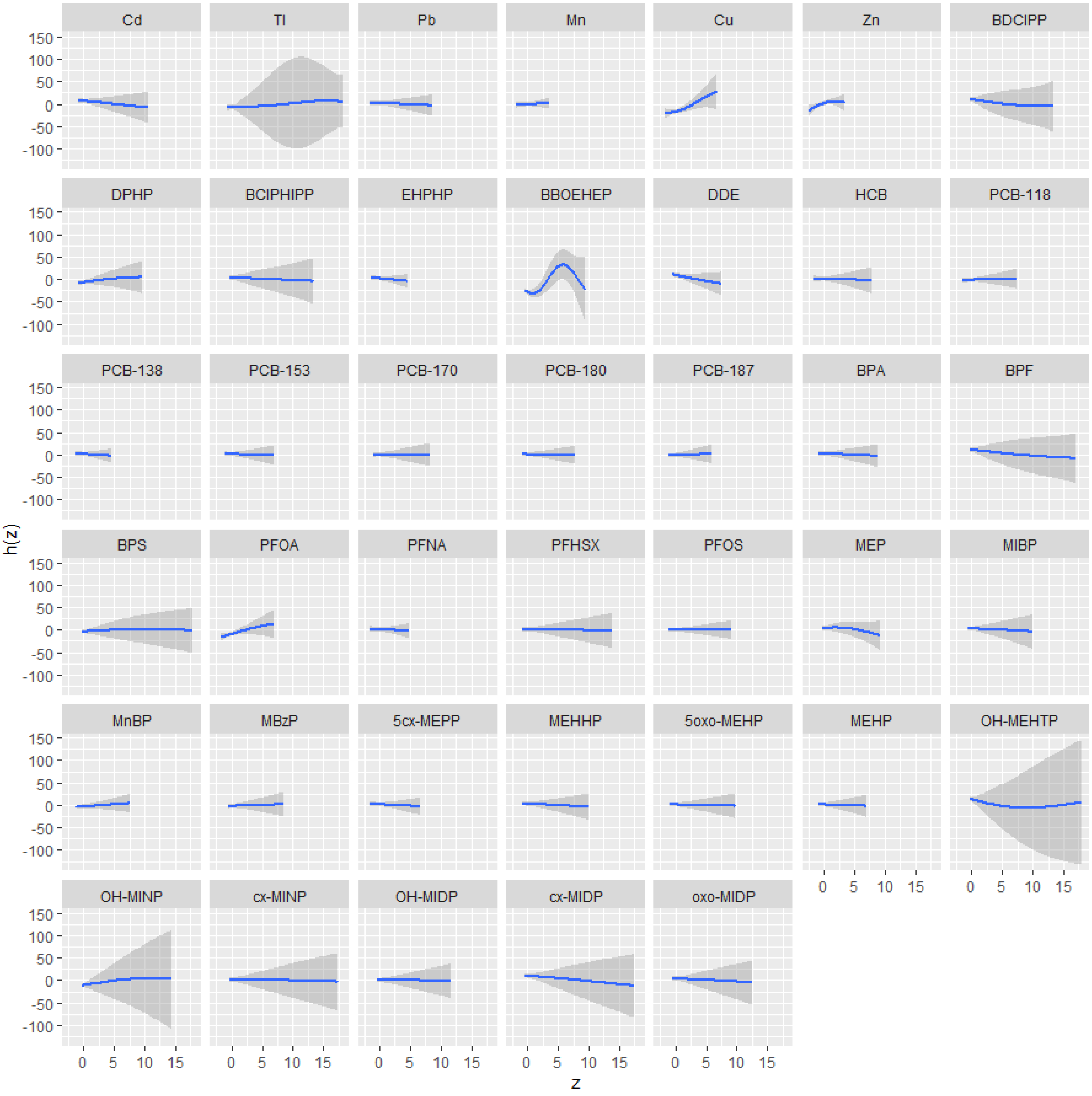

C

Figure S3 continued.

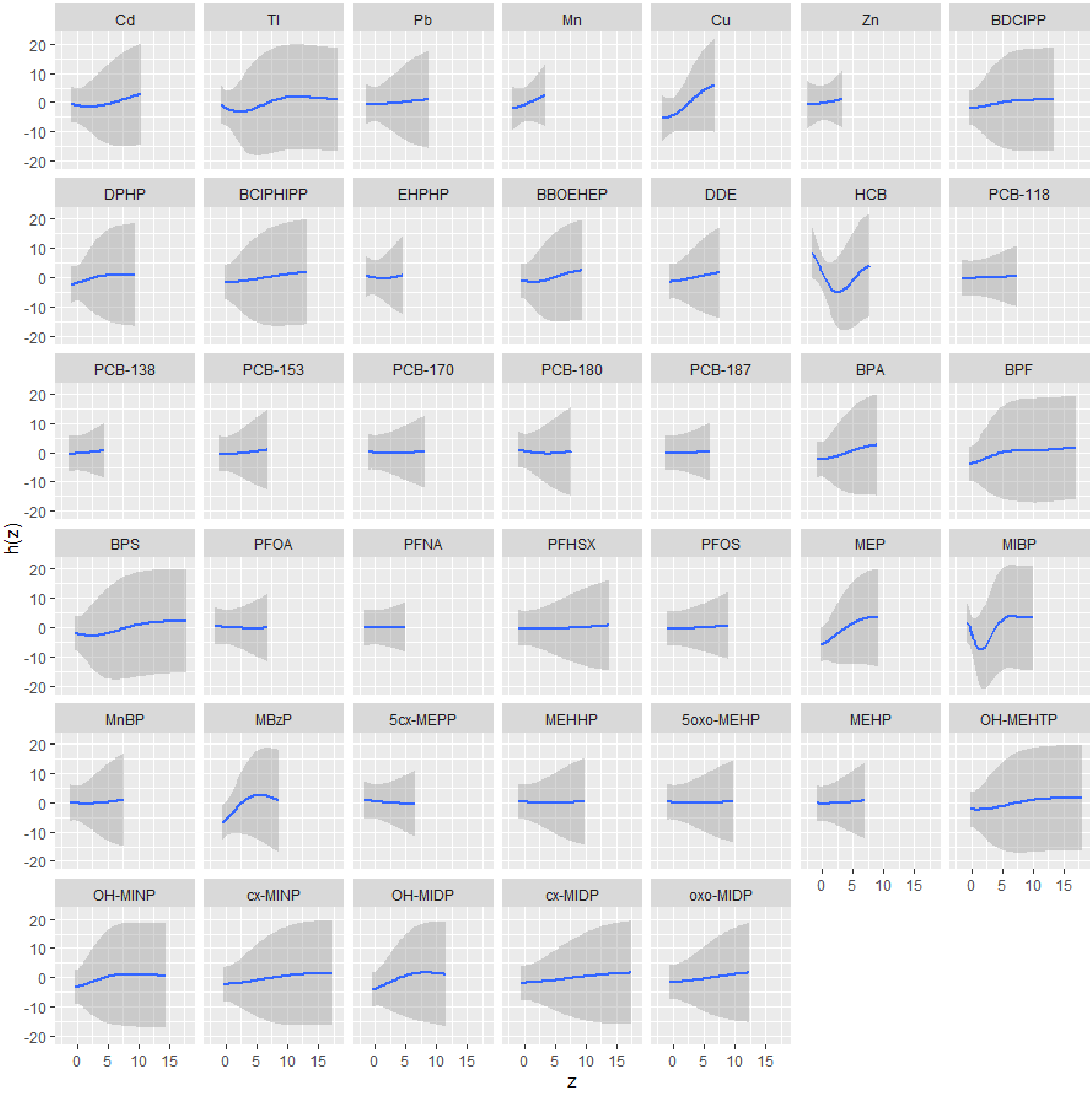

D

Figure S3 continued.

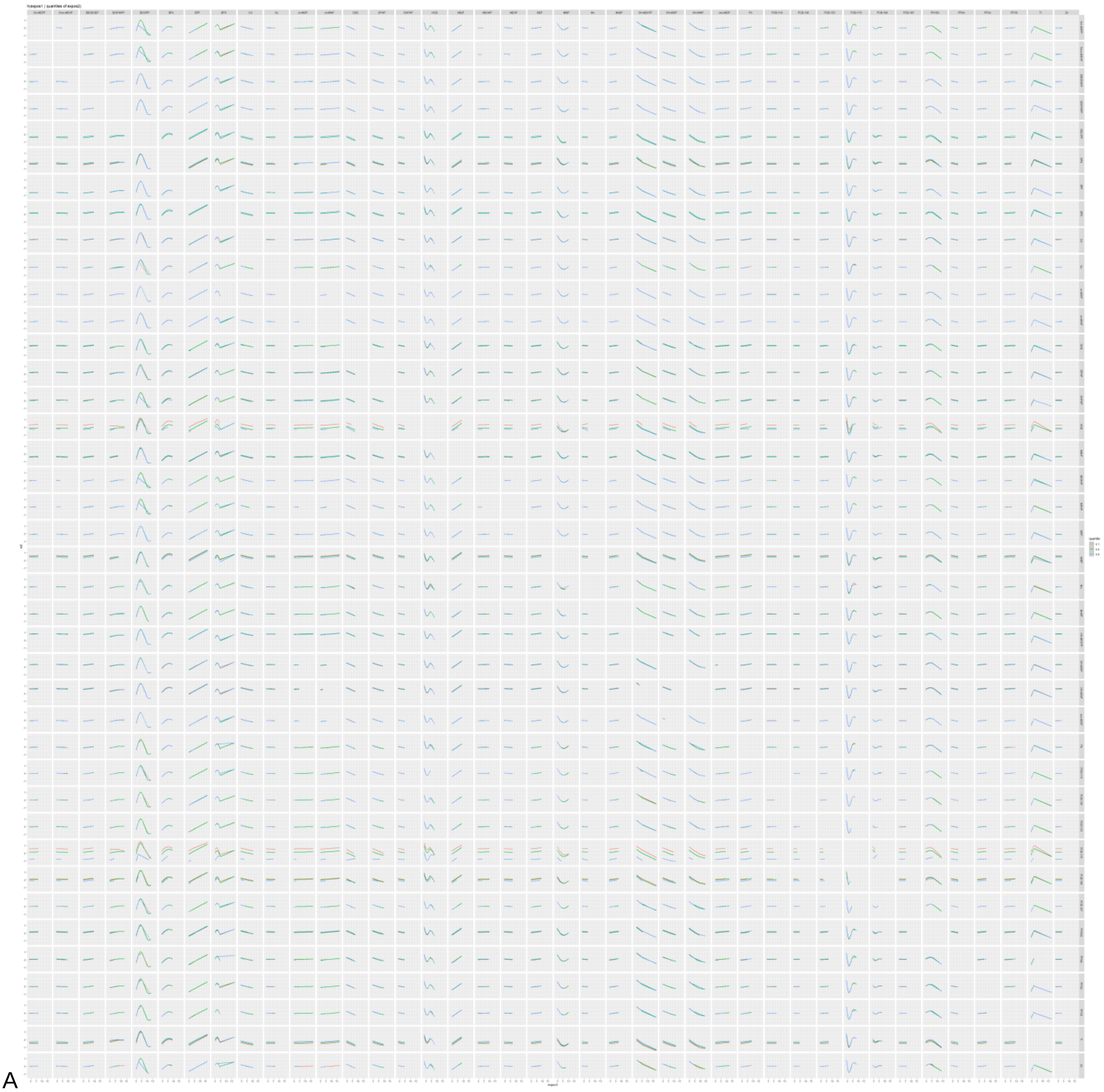

A

Figure S4. Qualitative interaction assessment between exposure 1 and exposure 2 in response to metabolic outcomes [A: body mass index z-score (zBMI), B: abdominal obesity (AO), C: cholesterol (TC) levels, D: triglycerides (TG) levels], estimated from Bayesian kernel machine regression (BKMR).
Note: models were adjusted for sex (except when outcome was zBMI), age (except when outcome was zBMI), sampling season, ever breastfed, highest education in the household, and physical activity.

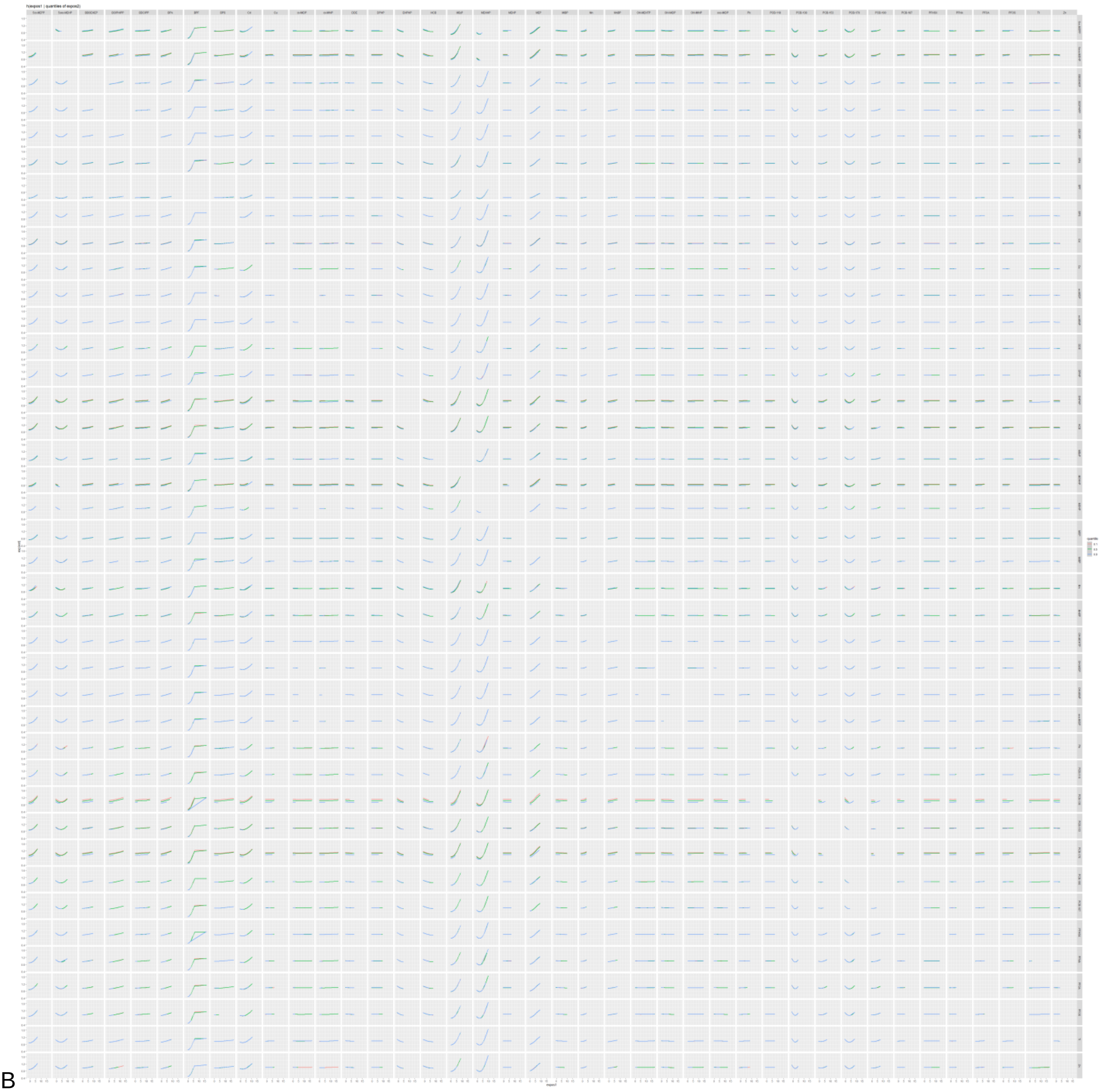

B

Figure S4 continued.

C

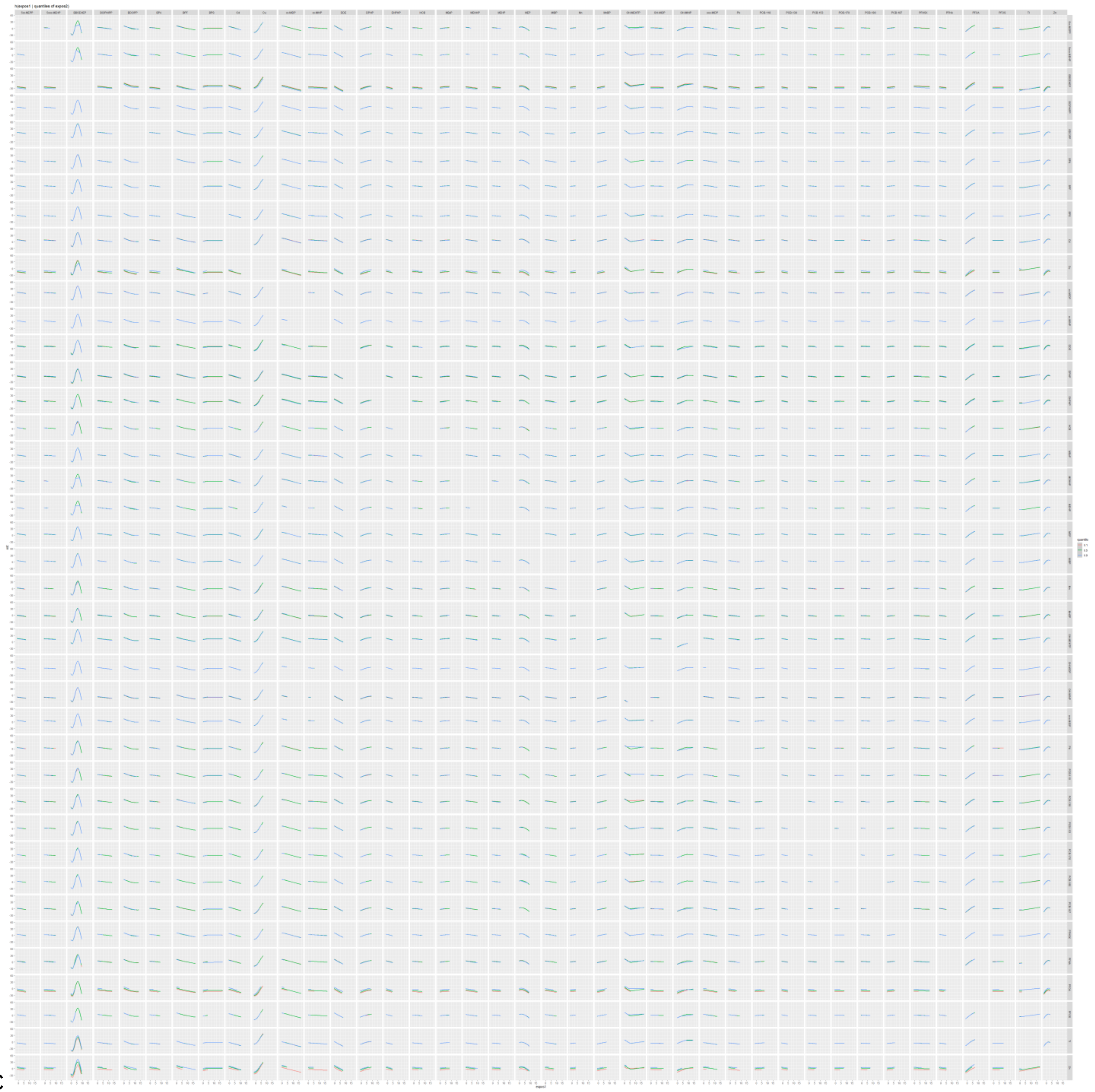

Figure S4 continued.

D

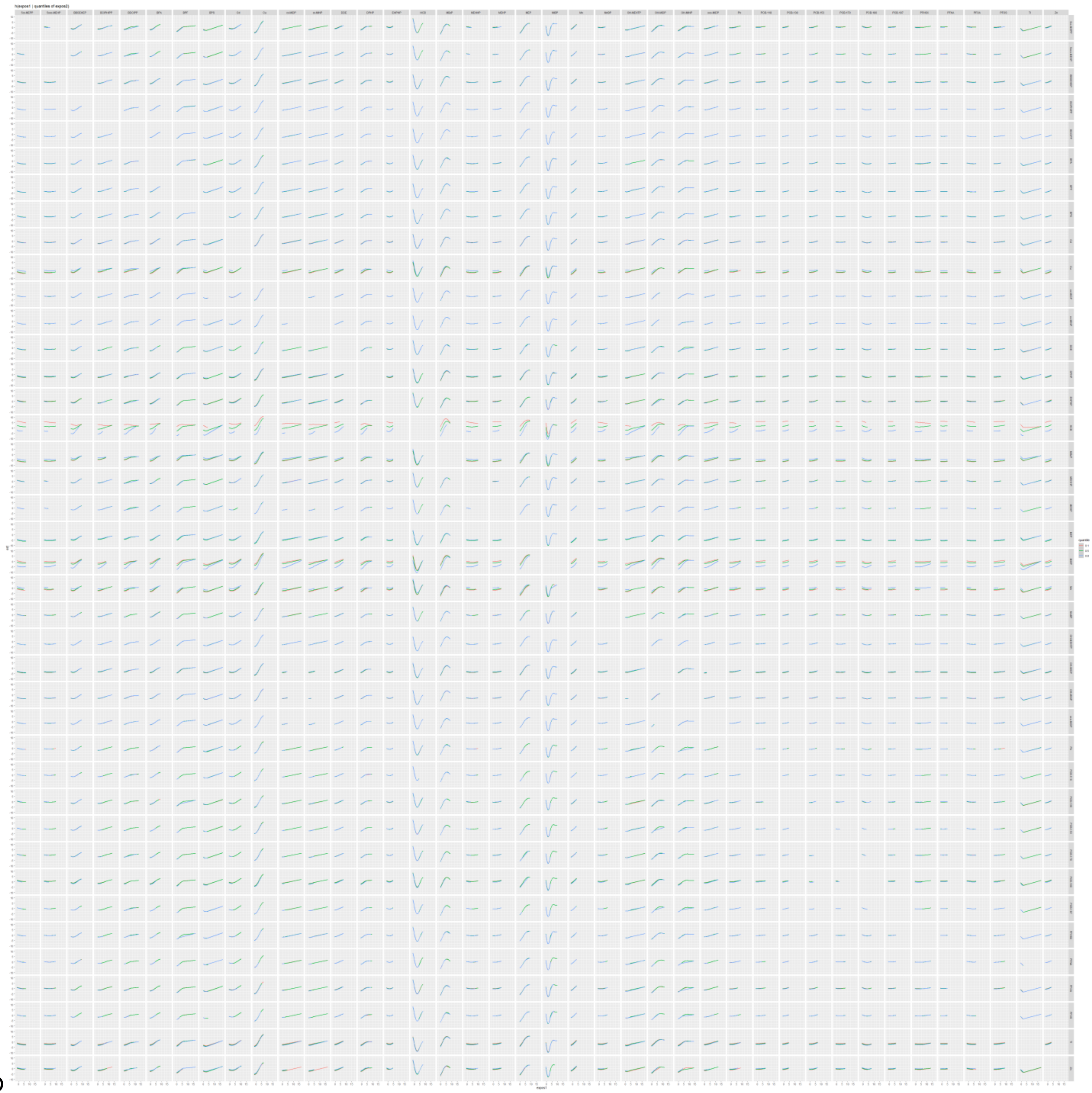

Figure S4 continued.
